# Isolation and characterization of microplastics from human blood samples by confocal RAMAN microscopy^☆^

**DOI:** 10.1016/j.mex.2026.103841

**Published:** 2026-02-25

**Authors:** Antonio José Sarabia, Belén Martínez, María de los Ángeles Martínez, Ricardo Rivera, María Isabel Torres, Antonio Peñas, Jorge Nicolás Domínguez

**Affiliations:** a Department of Experimental Sciences, Faculty of Experimental Sciences, University of Jaén, Jaén 23071, Spain; b Department of Inorganic and Organic Chemistry, Faculty of Experimental Sciences, University of Jaén, Jaén 23071, Spain; c Intensive Care Unit, University Hospital of Jaén, Jaén 23007, Spain; d Centro de Investigación Biomédica en Red de Enfermedades Cardiovasculares (CIBERCV), Spain

**Keywords:** Microplastics, Human, Blood, Confocal RAMAN microscopy

## Abstract

Microplastics (MPs) and nanoplastics (NPs) are emerging environmental contaminants increasingly detected in human tissues and fluids, highlighting the need for reliable analytical methods capable of isolating and characterizing these particles in complex biological matrices while reducing contamination risks. This work presents a systematic, integrative, and reproducible protocol for detecting MPs in human blood using confocal Raman microscopy. The method incorporates strict contamination-control measures, includes negative and positive controls to ensure analytical reliability, and provides reference Raman spectra from commonly used clinical and laboratory materials to identify potential sources of cross-contamination. Spectral data are compared using the open-source platform Open Specy, enabling similarity matching with an extensive polymer database and improving the confidence of particle identification. Application of the protocol enabled the detection and characterization of MPs in human blood samples, identifying polymers such as polystyrene (PS), ethylene-vinyl acetate (EVA), and polyethylene (PE). Overall, this protocol demonstrates high specificity for detecting MPs in human blood and provides a robust framework for future exposure studies.

• Provides a contamination-controlled analytical framework for MP detection in human blood.
• Integrates reference materials and controls to ensure data reliability and trace contamination sources.
• Uses open-source spectral comparison to support confident polymer identification.

## Graphical abstract

Schematic workflow of the proposed three-step protocol for the isolation and characterization of microplastics (MPs) in human blood. The procedure integrates: (1) MPs decontamination and quality control, including the use of negative controls (NC), plastic-free materials, and contamination-minimization measures; (2) sample processing, involving blood collection, digestion, and filtration to retain MPs (<5 mm); and (3) MPs identification by confocal Raman microscopy, enabling reliable detection and classification through comparison with spectral libraries. This approach highlights the simplicity, reproducibility, and contamination-minimized nature of the method. Figure created with BioRender.com.

**Figure:**
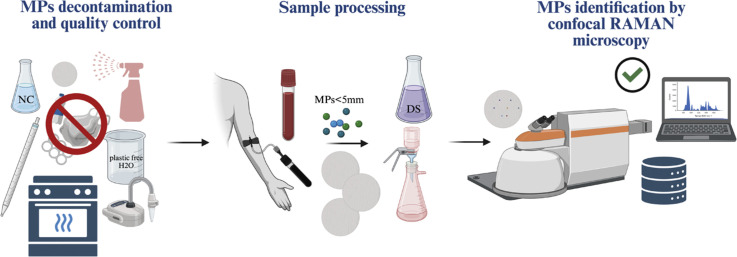

**Specifications table**

**Table:** 

**Subject area**	Chemistry
**More specific subject area**	*Raman spectroscopy applications in biomedical research*
**Name of your protocol**	*Three-steps protocol for MPs identification in human blood*
**Reagents/tools**	*1. Ethanol 70 %*
	*2. Distilled water*
	*3. Ultrapure water (glass filtered)*
	*4. Potassium hydroxide (KOH, 10 %)*
	*5. Tween-20 (Polysorbate-20, 1 %)*
	*6. Nitric acid (HNO_3_, 67 %)*
	*7. Methanol (MeOH)*
	*8. Glass fiber filter membranes (diameter 25mm, nominal pore size (∼1.6µm), Whatman; WHA1820025)*
	*9. Kugelrohr glass oven (BUCHI 046600)*
	*10. Glass Petri dishes*
	*11. Glass flask*
	*12. Glass beakers*
	*13. 15- and 40-mL glass vials*
	*14. Glass pipettes*
	*15. Stainless steel forceps*
	*16. Aluminium foil*
	*17. Environmental aspirator (LSRFXF)*
	*18. Cotton lab coats*
	*19. Double sided adhesive tape*
	*20. Glass Petri dishes*
	*21. Standard commercial polyethylene*
	*22. Raman inVia de Renishaw*
	*23. Open Specy free platform*
**Experimental design**	*Human blood samples were collected under contamination-controlled conditions, digested and filtered to isolate retained particles, and subsequently analyzed by confocal Raman microscopy using Open Specy for polymer identification.*
**Trial registration**	*Not applicable*
**Ethics**	*All human blood samples were obtained from voluntary donors after written informed consent. The study was conducted in accordance with the Declaration of Helsinki and approved by the appropriate institutional ethics committee.*
**Value of the Protocol**	*- Provides a standardized workflow for isolating and characterizing microplastics (MPs) in human blood, from sample collection to Raman-based identification.*
	*- Minimizes contamination risks through integrated decontamination and quality control steps, ensuring more reliable and reproducible results.*
	*- Offers a simple and cost-effectiveapproach that can be widely adopted to support future biomedical and environmental health studies.*

## Background

Global plastic production has increased from 1.5 million tons to approximately 359 million tons in the last 70 years [1], and it is expected to reach 500 million tons by 2025 [2]. Forward-looking analyses indicate that under current trends, production may exceed 1 billion tons annually by 2050, highlighting the increasing environmental burden of plastics and associated microplastics [3]. However, no >10 % of plastic waste is recycled [4], and most of the waste is incinerated, disposed of in landfills, or directly discharged into the environment, becoming a major source of microplastics (MPs) and nanoplastics (NPs) [5].

MPs and NPs are plastic particles smaller than 5 mm and 1 μm in diameter, respectively [6,7]. They have raised increasing concern due to their omnipresence in the environment and their potential impacts on human health, becoming a significant environmental issue in recent decades. These particles, derived from the degradation of larger plastic products such as tire wear, textile fibers, and personal care products, have been identified in virtually all ecosystems, from oceans to agricultural soils, freshwater systems, and even the air we breathe [6,8]. Due to their low density, MPs and NPs can be transported between terrestrial and aquatic ecosystems by wind and water currents, facilitating global dispersal. Consequently, MPs and NPs may enter the human body through ingestion, inhalation, and dermal contact, mainly via contaminated water, seafood, and other food sources, with potential accumulation in biological tissues and associated health risks [9].

The most common forms of plastics are resins and fibers, including polyethylene (PE), polypropylene (PP), polystyrene (PS), polyvinyl chloride (PVC), polyethylene terephthalate (PET), and polyurethane (PUR) resins, along with polyester, polyamide, and acrylic (PP&A) fibers [10,11]. Once released, MPs and NPs can be transported over long distances by water currents and wind, facilitating their global dispersal [12]. This widespread distribution facilitates their entry into aquatic ecosystems, raising serious questions about their impacts on biodiversity and the health of living organisms [13].

In line with this, MPs and NPs represent a growing threat with potential repercussions throughout the food chain [14] and, consequently, a negative impact on human health [15]. Human exposure to MPs and NPs occurs mainly through ingestion, inhalation, and dermal contact, leading to their accumulation in various organs, tissues, and body fluids [16,17]. These particles have been detected in human liver, kidneys, brain, lungs, heart, spleen, and placenta, suggesting their ability to infiltrate and persist in vital systems [[18], [19], [20]]. Evidence indicates that MPs and NPs can induce oxidative stress, inflammation, and endocrine disruption [17,21,22], while available evidence in humans, still limited and largely observational, includes associations with respiratory, digestive and nervous alterations [23], as well as recent clinical observations linking the presence of MPs in atherosclerotic plaques with an increased risk of cardiovascular events, including myocardial infarction and ischemic stroke [24,25].

**Fig. 1:**
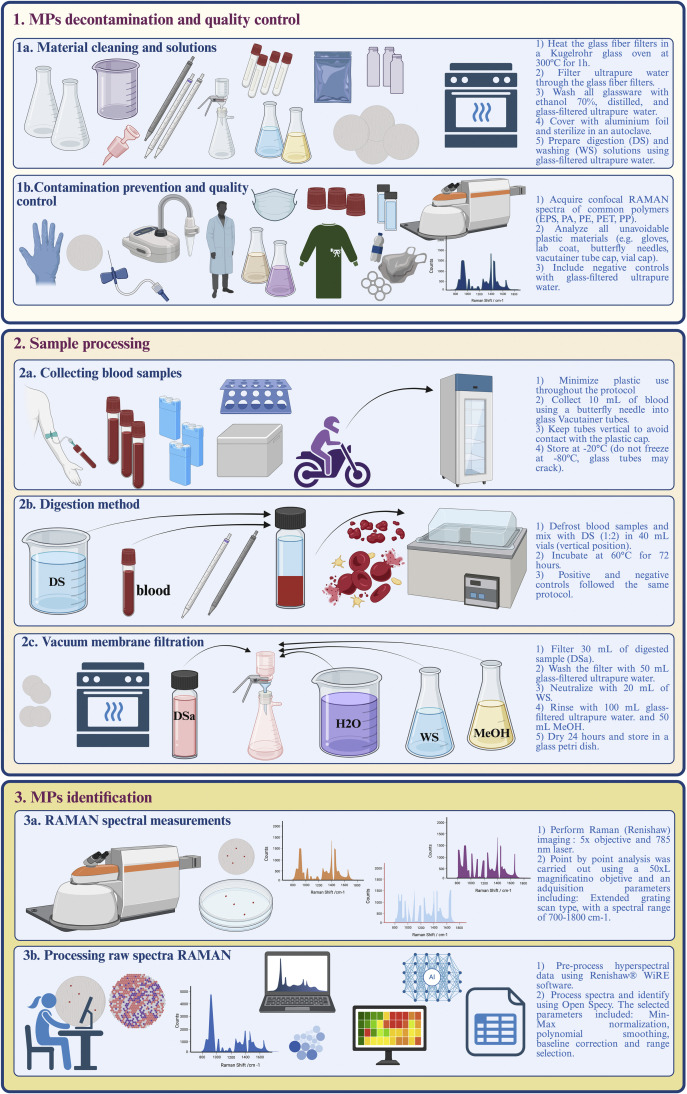
Workflow of the three-step protocol for isolating and analyzing microplastics (MPs) from human blood samples. The workflow comprises: (1) MP decontamination and quality control, including (1a) material cleaning and solution preparation and (1b) contamination prevention and quality control; (2) sample processing, including (2a) blood collection, (2b) digestion, and (2c) vacuum membrane filtration; and (3) MP identification, consisting of (3a) Raman spectral measurements and (3b) processing of raw Raman spectra.

Given this evidence of human exposure and potential health risks, the isolation and analysis of MPs and NPs in human biological samples is of particular interest. Although some studies have described methods to isolate MPs and NPs from tissues [19,[26], [27], [28]] or human blood using enzymatic, acidic, or alkaline digestion [[29], [30], [31]], there is still a lack of a straightforward, cost-effective, and reproducible protocol that minimizes contamination and covers the entire process, from sample collection to MPs identification. This study presents a systematic and integrative protocol for the isolation and characterization of MPs from human blood samples using confocal Raman microscopy. This method incorporates practical steps and technical recommendations to reduce plastic contamination in the surrounding environment, while preserving the structural integrity of MPs. This approach is integrated into a three-step protocol that enhances clarity and reproducibility: 1) MPs decontamination and quality control, 2) Sample processing and, 3) MPs identification**.** For practical use, a detailed workflow diagram is provided (Fig. 1) that summarizes the procedure.

## Description of protocol

Blood samples were collected at the Intensive Care Unit of the Hospital Universitario de Jaén, Jaén (Spain) in accordance with the Helsinki guidelines, ensuring proper human care and ethical standards. Research permits were obtained from Universidad de Jaén Ethics Committee (license code: CIOMGAB_20221121bis) and Provincial Research Ethics Committee of Jaén (license code: SICEIA-2025–000070). Written informed consent was obtained from all subjects before blood sampling.

**1. MPs decontamination and quality control**

*1a. Material cleaning and solutions*

- Glass fiber filter membranes (diameter 25 mm, nominal pore size (∼1.6 µm), Whatman; WHA1820025) were pre-treated at 300 °C for 1 h using a Kugelrohr glass oven under vacuum (Model BUCHI 046600). The vacuum environment facilitates the decomposition and removal of polymer-derived contaminants while avoiding thermo-oxidative carbonization, and additionally minimizes exposure to ambient MPs contamination by eliminating the need for carrier gases.

**Note:** After heating, store the filters on a clean glass Petri dish (all in the same orientation), ready for use.

- Filter ultrapure water by using the glass fiber filter membrane.

- All material (flasks, glass Petri dish, beakers, 15- and 40-ml glass vials, glass pipettes, forceps) was cleaned before and after any practice following the next step:

1 First, wash with running water and a small amount of ethanol 70 %.
2 Rinse with distilled water.
3 Rinse with glass-filtered water.
4 Finally, cover glass material with aluminium foil and sterilize them in an autoclave.

- All solutions used in the protocol were made with glass-filtered water and filtered by a glass filter.

**Note:** This process removes any microplastics contamination from all materials.

*1b Contamination prevention and quality control*

**Fig. 2:**
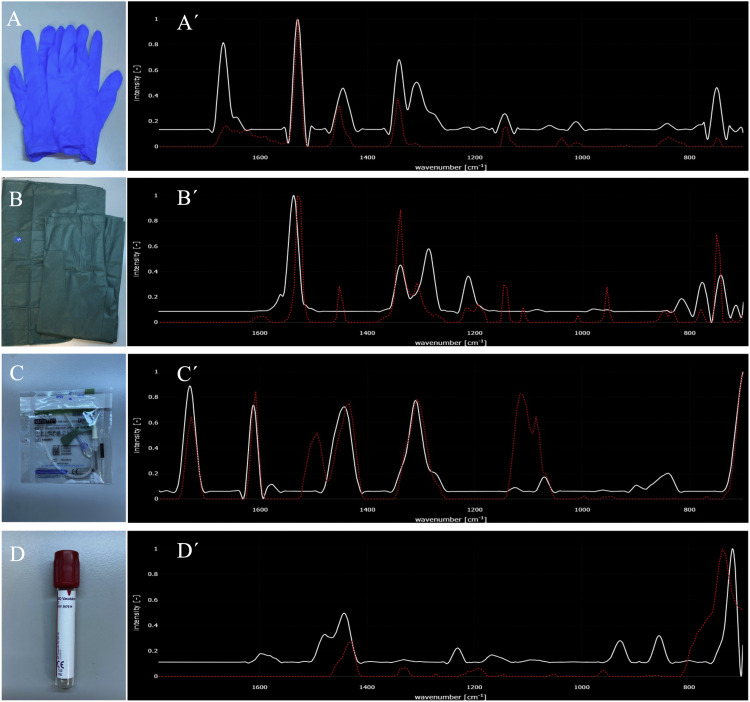
Confocal Raman analysis of potential contamination sources. (A) Nitrile gloves (polyhaloolefin, PE and PP), (B) clinical clothing (polypropylene, PP), (C) butterfly needle cannula (polyhaloolefin, PE and PP), and (D) Vacutainer glass tube cap (polytetrafluoroethylene, PTFE). Panels A′–D′ display the Raman spectra obtained for each material, compared with reference spectra from Open Specy, a free online platform for spectral analysis. Additional laboratory items analyzed, together with their polymer identification, are summarized in Table 1. These spectra were used as reference controls to identify and account for potential cross-contamination during sample collection and processing.

To minimize the risk of plastic cross-contamination, the use of plastic materials was reduced as much as possible at every step of this protocol. The only stage where blood was in contact with some plastic surface, for a short period of time, was during extraction. This was due to the use of plastic vacuette safety butterfly needles (21Gx19cm + luer adapter, sterile; Greiner KG450081), which are the only approved devices for blood collection (Fig. 2). Additionally, a small aspirator (LSRFXF) was used to help reduce contamination from suspended particles in the environment. To further minimize airborne microplastic contamination, all procedures were carried out under controlled laboratory conditions, ensuring that the workspace remained free of synthetic fibers by using cotton laboratory coats and by routinely cleaning surfaces before sample handling.

Furthermore, all instances, both in hospital and laboratory settings, where the use of plastic materials was unavoidable were taken into account. Accordingly, every plastic material used at any stage of the protocol was analysed to obtain confocal-Raman spectra (Fig. 2; Table 1).

**Table 1:** Identification of polymers in common laboratory items using Raman spectroscopy and analysis with Open Specy, a free online platform for spectral comparison. The table lists the items tested and their corresponding polymer types. Note that the laboratory coat used during the protocol is described in the Reagents/Tools section (100 % cotton lab coat), whereas the PET laboratory coat listed here represents a common laboratory garment included as a potential source of environmental contamination.

Laboratory items	Identified polymer (Open Specy)	Similarity %
Lab coat	Polyethylene terephthalate (PET)	97
Clinical pyjama	Polypropylene (PP)	54
Nitrile gloves	Polyolefin (PE and PP)	76
Latex gloves	Polydiene (butadiene, isoprene)	95
Butterfly needle	Polyolefin (PE and PP)	73
Vacutainer tube cap	Polytetrafluoroethylene (PTFE)	52
Bottle of KOH pellets	Polyethylene (PE)	96
Inner plastic of the filter box	Polystyrene (PS)	96
Double sided adhesive tape	Polypropylene (PP)	91
Inner part of the vials cap	Polydimethylsiloxane (PDMS)	98

**2. Sample processing**

*2a. Collecting blood samples*

− Disinfect the area before proceeding with venipuncture.
− Collect 10 ml of blood into two 5 mL glass Vacutainer tubes (BD *Vacutainer 367614*), with a vacuette safety butterfly needle (21Gx19 cm + luer adapter, sterile; Greiner KG450081).
− Keep the glass tubes, containing blood, in a vertical position to prevent contact between blood and the plastic cap, and store at 4 °C during transport to the laboratory.

**Note:** Blood collecting should be performed by clinical staff.

- Transfer the glass vacutainer tube containing the blood sample as soon as possible into a 15 mL vial and store it in a freezer at −20 °C until the next step.

**Note:** Glass Vacutainer tubes do not resist low temperature and glass will fissure. In case the Vacutainer tube breaks, the vial will still contain the blood during the defrost process.

*2b Digestion method*

− Prepare the digestion solution (DS): 10 % KOH+1 % Tween-20.
− Defrost the 15 mL vials containing the glass Vacutainer tube, in which blood is stored.

**Note:** DS ensures effective tissue digestion while minimizing physical or chemical degradation of the most common MPs (PE, PP, PS). It is also important to measure the initial blood volume, as this allows the results to be expressed as the number of particles per milliliter of blood.

− Add 2 vol of DS for each 1 vol of blood (e.g., 20 mL DS; 10 mL blood) in 40 mL vials.
− Keep the tubes in a vertical position and incubate in a bath at 60 °C for 72 h. No shaking is required.

**Note:** Avoid covering the tubes with aluminium foil; aluminium foil is highly reactive with bases. Silicone is not affected by DS.

*2c.Vacuum membrane filtration*

− Assemble the glass fiber filter (previous decontamination) before filtration.
− Filter the digested biological sample (30 mL).

**Note:** After filtration, all volume of the sample is discarded.

− Wash the glass fiber filter membrane with 50 mL of glass-filtered water.
− Neutralize the glass filter (without vacuum) with 20 mL of wash solution (WS) (67 % HNO_3_ + 1 % Tween-20).

**Note:** This step was performed without vacuum to enhance the neutralization reaction. It is important to prepare WS at the time of use.

− Wash it with glass-filtered water (100 mL).
− Dehydrate the filter with MeOH (50 mL).
− Dry it at room temperature for 24 h.

**Note:** Once the filter is dry, keep the membrane covered in a glass Petri dish until analysis by confocal-RAMAN.

**3. MPs identification**

*3a. RAMAN spectral measurements*

- Set up the filter on the top lid and stick it using double-sided adhesive tape. Finally cover it with the bottom lid.

**Note:** This is due to the bottom lid hit with the 50xL Raman objective.

- Make a mark on the edge of the filter to indicate its orientation to facilitate subsequent analysis.

**Note:** Avoid labelling with marker, pencil or pen

- *Prepare equipment:*

1 Turn on the computer and the 785 nm laser.
2 Open the WiRE software and confirm that the laser is associated with the correct monochromator (1200I/mm).

- *Place and focus the sample:*

1 Press the “Door Release” button before opening the microscope door.
2 Place the filter (with the bottom lid) in the microscope stage.
3 Active the camera icon to visualize the filter
4 Select the 5x objective lens both on the microscope and in the WiRE software.
5 Focus manually to view clearly the surface of the filter.
6 In the “Live Video” window, select “Set Origin” to set the centre of the filter as the (0,0) coordinate.

- *Filter Image Acquisition:*

1 In the “Live video” window, select “Snap → Montage”.
2 Define manually the limits by entering (−6000, 6000) for both X and Y axes.
3 Close the microscope door and press “Run” to capture the image.
4 Save it.

- *Particle Image Acquisition:*

1 In the “Live video” window, select “Snap → Single”.
2 Save it.

**Note:** Raman microspectroscopic imaging was performed through a Renishaw Centrus 24QW69 detector and a 5x magnification objective.

- *Laser Calibration:*

1 After the laser has warmed up for 10 min, go to “Tools Quick Calibration”.
2 Confirm that a peak appears at 520 nm with an intensity of approximately 30,000 counts.
3 Close the calibration window.

- *Point by point analysis:*

1 Lower the stage using the coarse focus knob, without moving the sample.
2 Switch to the 50xL objective on both the microscope and the WiRE software.

**Note:** The 50xL magnification objective indicates a long working distance (“L”).

3 Locate the particle of interest and focus it.
4 Capture an image of the particle before the measurement. Go to “Live Video” → “Save Image”.

**Note:** Optionally, acquire a photo after the measurement to evaluate whether laser-induced changes occurred in the analysed particle.

5 In Measurements, click New → Spectral Acquisition. Acquisition parameters:
  • Grating scan type: Extended
  • Spectral range: 700–1800 cm⁻¹
  • Exposure time: 10 s *(mandatory for Extended mode)*
  • Laser power: 0.1–1 %
  • Accumulations: 5–20
  **Note:** Adjust acquisition parameters (exposure time, laser power, accumulations) based on the particle’s properties. Especially if the signal is low or the particle shows signs of degradation.
6 Press the RUN button.
7 Selection of random points for analysis:
  • Divide the filter into 4 quadrants.
  • Coordinates were randomly generated using an AI tool.
  • If there is no visible particle at a chosen point, define a consistent method to select the nearest one.

**Note:** The analysis was designed to target and characterize random particles. To minimize the risk of potential photo-damage to the sample, an appropriate ratio between laser power and exposure time was ensured.

*3b Processing raw spectra RAMAN*

Raman spectroscopic analysis was performed to identify polymer particles using the open-source platform Open Specy [32], which compares experimental Raman spectra with a comprehensive plastic database.

The analysis was carried out under the following standardized parameters:

*Pre-processing:*

1 Min–Max normalization
2 Smoothing with a 5th order polynomial (28)
3 Derivative function: disabled (set to 0)
4 Wavenumber window: 50 cm⁻¹
5 Baseline correction: polynomial order 5 with 25 iterations
6 Spectral range: 700–1800 cm⁻¹

*Spectral identification:*

1 Spectrum type: Raman
2 Transformation: no baseline
3 Identification method: Full (search across the complete library)
4 Correlation threshold: 0.7

These parameters allowed the reliable identification of several common polymers present in the analyzed samples.

## Protocol validation

Using the proposed protocol, MPs were successfully isolated and analysed from human blood samples collected in a clinical setting. To assess potential sources of cross-contamination, several laboratory materials commonly used during sample collection and handling were tested by confocal Raman spectroscopy. The main items illustrated in Fig. 2 include nitrile gloves (Fig. 2A), clinical clothing (Fig. 2B), the butterfly needle cannula (Fig. 2C), and a Vacutainer glass tube (Fig. 2D), with their corresponding Raman spectra shown in panels A′–D′. For each material, representative Raman spectra were obtained and compared with reference data available in Open Specy, a free online platform for spectral analysis, to confirm their polymer composition. These materials were analysed because any contamination derived from them would be visible in the negative controls, thereby ensuring that MPs identified in blood samples could be distinguished from procedural artefacts. Additional items tested, together with their identified polymers and similarity percentages, are summarised in Table 1, which was used to compile a reference library of unavoidable laboratory-derived materials for contamination control.

**Fig. 3:**
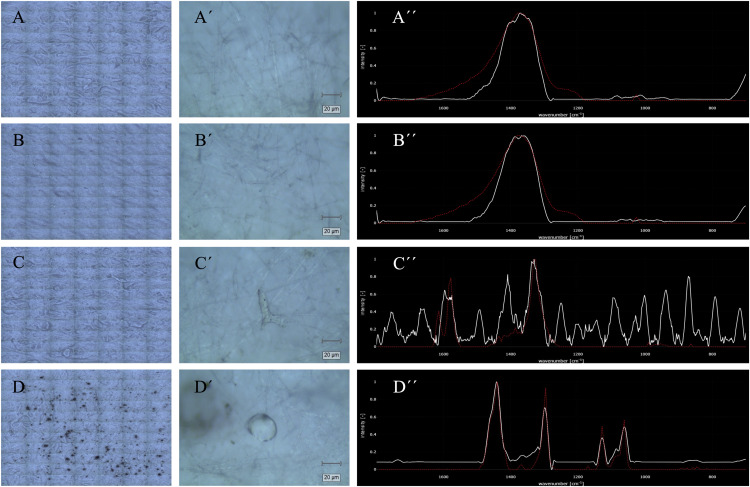
Negative and positive controls analyzed by confocal Raman spectroscopy. (A) Ultrapure water filtered through a glass fiber filter. (B) Ultrapure water filtered through a glass fiber filter and passed through a butterfly needle. (C) Digestion solution prepared using glass-filtered ultrapure water, where crystalline KOH structures can be observed. (D) Positive control consisting of a commercial plastic standard (polyethylene). (A–D) Images of the filters; (A′–D′) images of selected particles when present; and (A′′–D′′) Raman spectra (white) compared with reference spectra from Open Specy (red). No spectra from negative controls (A–C) could be matched in the Open Specy database, confirming the absence of detectable microplastic particles. In contrast, the positive control (D) yielded a spectrum that matched the commercial polyethylene standard.

Negative and positive controls were included to evaluate the reliability of the protocol and to rule out potential contamination from laboratory materials or solutions. The negative controls were designed to detect any artefactual signals that might arise during sample handling: ultrapure water filtered through a glass fiber filter (Fig. 3A), ultrapure water filtered through a glass fiber filter and passed through a butterfly needle (Fig. 3B), and digestion solution prepared with glass-filtered ultrapure water (Fig. 3C). Moreover, a positive control was included to validate the performance of the protocol, consisting of a standard commercial polyethylene (REPSOL Alcudio 1940C) (Fig. 3D). In each case, the filter images are presented on the left (Fig. 3A–D), while the central panels (Fig. 3A′–D′) display the selected particle when present; the Raman spectra are shown on the right (Fig. 3A′′–D′′). No identifiable spectra from the negative controls (Fig. 3A–C) matched any reference in the Open Specy database, confirming the absence of MPs signals. In the DS control (Fig. 3C), crystalline structures consistent with KOH are visible, but none showed spectra compatible with plastic polymers. Conversely, the positive control (Fig. 3D) produced a spectrum with similarity to the polyethylene standard, thus demonstrating the validity of the method.

**Fig. 4:**
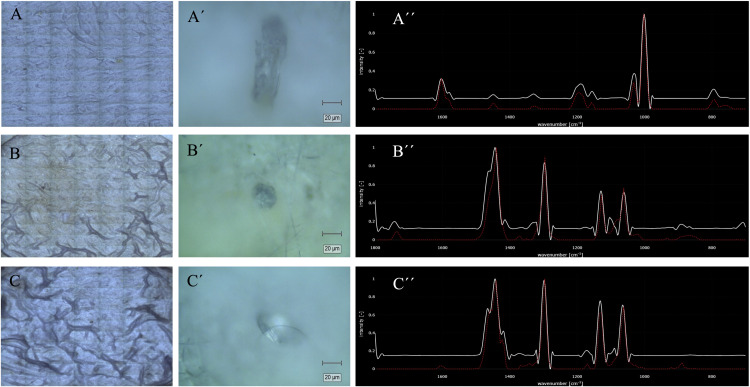
Confocal Raman analysis of MPs isolated from human blood samples. Panels A, B, and C correspond to three independent blood samples. (A–C) Images of the filters after the isolation process. (A′–C′) Images of individual particles selected for analysis. (A′′–C′′) Raman spectra of the particles (white), compared with reference spectra in the Open Specy database (red). The software provided similarity matches with polystyrene (PS) in sample A, ethylene-vinyl acetate (EVA) in sample B, and polyethylene (PE) in sample C.

To confirm the presence and identity of MP particles, blood samples were analysed using confocal Raman spectroscopy. Representative results from three independent samples are shown in Fig. 4. For each sample, the left panels (Fig. 4A–C) display the filter images after the isolation process; the middle panels (Fig. 4A′–C′) show the corresponding particle images selected for analysis; and the right panels (Fig. 4A′′–C′′) present the Raman spectra of those particles. The spectra were compared with reference spectra using the Open Specy database, which provided matches to polystyrene (PS) for sample A, ethylene-vinyl acetate (EVA) for sample B, and polyethylene (PE) for sample C. These results illustrate the ability of confocal Raman spectroscopy to detect and identify microplastics in human blood samples with high specificity.

## Limitations

Despite the robustness and reproducibility of the proposed protocol, certain limitations inherent to Raman microscopy should be acknowledged. First, the practical lower size limit for reliable MP identification is approximately 2–3 μm; below this threshold, or in the presence of strong fluorescence, the signal-to-noise ratio often compromises spectral quality and matching confidence. Second, due to time constraints, it is not feasible to analyse the entire filter surface, and a representative subset of particles is therefore examined. To minimize potential sampling bias—particularly relevant in samples with low particle density—a randomized grid-based selection strategy was employed. While sub-sampling is a standard requirement in Raman-based analyses, the subsample size and analysis time were selected to be sufficient to reflect the overall polymer composition. Nevertheless, very rare polymer types may remain underrepresented in low-density samples. Additionally, baseline-correction algorithms can occasionally introduce artefacts in low-quality spectra, which were carefully monitored to avoid misidentification.

Digestion and filtration may also slightly modify the appearance of glass fiber filters, increasing background scattering and generating less defined spectra—an effect commonly observed in Raman analyses of complex matrices. This highlights the importance of continued refinement and expansion of spectral libraries, as well as the incorporation of complementary approaches to ensure accurate identification in challenging samples.

Overall, the protocol provides a solid and practical framework for detecting and characterizing MPs in human blood, and these considerations mainly reflect intrinsic analytical challenges rather than limitations of the methodology itself.

## CRediT author statement

**Antonio José Sarabia:** Investigation, Visualization**,** Data Curation, Writing - Original Draft. **Belén Martínez:** Investigation. **María de los Ángeles Martínez:** Resources, Investigation. **Ricardo Rivera Fernández:** Resources, Investigation.**María Isabel Torres:** Writing – Review & Editing, Supervision. **Antonio Peñas:** Methodology, Validation, Writing – Review & Editing. **Jorge Nicolás Domínguez:** Conceptualization, Methodology, Writing – Review & Editing, Supervision, Project administration, Funding acquisition.

## Declaration of generative AI and AI-assisted technologies in the writing process

During the preparation of this work, the authors used ChatGPT (GPT-5, OpenAI) to improve the readability, clarity of grammar, and to assist with translation from Spanish to English. After using this tool, the authors reviewed and edited the content as needed, and take full responsibility for the content of the published article.

## Declaration of competing interest

The authors declare that they have no known competing financial interests or personal relationships that could have appeared to influence the work reported in this paper.

## Data Availability

No data was used for the research described in the article.

## Appendix Supplementary materials

**Supplementary material *and/or* additional information [OPTIONAL]** During the optimization of the protocol, the procedure was transitioned from using larger glass fiber filters (47 mm diameter, nominal pore size (∼1.6 µm), Whatman; WHA1825047) to smaller ones with the same pore size (25 mm diameter, nominal pore size (∼1.6 µm), Whatman; WHA1820025). This reduction in filter diameter allowed a higher concentration of retained MPs, thereby improving their recovery and subsequent analysis. Image, application 1

## References

[1] Bui X.T., Vo T.D.H., Nguyen P.T., Nguyen V.T., Dao T.S., Nguyen P.D. (2020). Microplastics pollution in wastewater: characteristics, occurrence and removal technologies. Environ. Technol. Innov..

[2] Huang D., Tao J., Cheng M., Deng R., Chen S., Yin L., Li R. (2021). Microplastics and nanoplastics in the environment: macroscopic transport and effects on creatures. J. Hazard. Mater..

[3] Khan H.S., Hasan J., Manik M., Farukh M.A., Shahjahan M. (2024). Pervasiveness of microplastics in the gastrointestinal tract of some selected fish species from Turag River alongside the capital city of Bangladesh. Emerg. Contam..

[4] Tumu K., Vorst K., Curtzwiler G. (2023). Global plastic waste recycling and extended producer responsibility laws. J. Environ. Manag..

[5] Osman A.I., Hosny M., Eltaweil A.S., Omar S., Elgarahy A.M., Farghali M., Yap P.S., Wu Y.S., Nagandran S., Batumalaie K., Gopinath S.C.B., John O.D., Sekar M., Saikia T., Karunanithi P., Hatta M.H.M., Akinyede K.A. (2023). Microplastic sources, formation, toxicity and remediation: a review. Environ. Chem. Lett..

[6] Jiang B., Kauffman A.E., Li L., McFee W., Cai B., Weinstein J., Lead J.R., Chatterjee S., Scott G.I., Xiao S. (2020). Health impacts of environmental contamination of micro- and nanoplastics: a review. Environ. Health Prev. Med..

[7] Galloway T.S., Cole M., Lewis C. (2017). Interactions of microplastic debris throughout the marine ecosystem. Nat. Ecol. Evol..

[8] Singh J., Samuel J., Hurley R. (2021). Editorial: plastics, microplastics, and nanoplastics: management and mitigation of water contamination. Front. Environ. Sci..

[9] Manik M., Hossain M.T., Pastorino P. (2025). Characterization and risk assessment of microplastics pollution in Mohamaya Lake, Bangladesh. J. Contam. Hydrol..

[10] Geyer R., Jambeck J.R., Law K.L. (2017). Production, use, and fate of all plastics ever made. Sci. Adv..

[11] PlasticsEurope. (2022). Plastics – the facts 2022 (Issue October), 2022.

[12] Liu L., Song J., Zhang M., Jiang W. (2021). Aggregation and deposition kinetics of polystyrene microplastics and nanoplastics in aquatic environment. Bull. Environ. Contam. Toxicol..

[13] Wang L., Wu W.M., Bolan N.S., Tsang D.C.W., Li Y., Qin M., Hou D. (2021). Environmental fate, toxicity and risk management strategies of nanoplastics in the environment: current status and future perspectives. J. Hazard. Mater..

[14] Foley C.J., Feiner Z.S., Malinich T.D., Höök T.O. (2018). A meta-analysis of the effects of exposure to microplastics on fish and aquatic invertebrates. Sci. Total Environ..

[15] Huang W., Song B., Liang J., Niu Q., Zeng G., Shen M., Deng J., Luo Y., Wen X., Zhang Y. (2021). Microplastics and associated contaminants in the aquatic environment: a review on their ecotoxicological effects, trophic transfer, and potential impacts to human health. J. Hazard. Mater..

[16] Nihart A.J., Garcia M.A., Hayek E.E., Liu R., Olewine M., Kingston J.D., Castillo E.F., Gullapalli. T. Howard R.R., Bleske B., Scott J., Gonzalez-Estrella J., Gross J.M., Spilde M., Adolphi N.L., Gallego D.F., Jarrell H.S., Dvorscak G., Zuluaga-Ruiz M.E., West A.B., Campen M.J. (2025). Bioaccumulation of microplastics in decedent human brains. Nat. Med..

[17] Rubio L., Marcos R., Hernández A. (2020). Potential adverse health effects of ingested micro- and nanoplastics on humans. Lessons learned from in vivo and in vitro mammalian models. J. Toxicol. Environ. Health - B: Crit. Rev..

[18] Verma K.K., Song X.-P., Xu L., Huang H.-R., Liang Q., Seth C.S., Li Y.-R. (2023). Nano-microplastic and agro-ecosystems: a mini-review. Front. Plant Sci..

[19] A. Ragusa, A. Svelato, C. Santacroce, P. Catalano, V. Notarstefano, O. Carnevali, F. Papa, M.C.A. Rongioletti, F. Baiocco, S. Draghi, E. D’Amore, D. Rinaldo, M. Matta, E. Giorgini, Plasticenta: first evidence of microplastics in human placenta, Environ. Int. (2021). 10.1016/j.envint.2020.106274. 10.1016/j.envint.2020.106274 33395930

[20] Prata J.C., da Costa J.P., Lopes I., Duarte A.C., Rocha-Santos T. (2020). Environmental exposure to microplastics: an overview on possible human health effects. Sci. Total Environ..

[21] Cho Y.M., Choi K.H. (2021). The current status of studies of human exposure assessment of microplastics and their health effects: a rapid systematic review. Environ. Anal. Health Toxicol..

[22] Rahman A., Yadav O., Sarkar A., Achari G., Slobodnik J. (2020). Environmental exposure to microplastics: a scoping review on potential human health effects and knowledge gaps. BLDE Univ. J. Health Sci..

[23] Otorkpa O.J., Otorkpa C.O. (2024). Health effects of microplastics and nanoplastics: review of published case reports. Environ. Anal. Health Toxicol..

[24] Zhang Y., Gao Q., Gao Q., Xu M., Fang N., Mu L., Han X., Yu H., Zhang S., Li Y., Gong Y. (2025). Microplastics and nanoplastics increase major adverse cardiac events in patients with myocardial infarction. J. Hazard. Mater..

[25] Marfella T.S.R., Prattichizzo F., Sardu C., Fulgenzi G., Graciotti L., D’Onofrio M.M.N., Scisciola L., La Grotta R., Frigé C., Pellegrini V., Siniscalchi G.A.M., Spinetti F., Vigliotti G., Vecchione C., Carrizzo A., Squillante A.F.A., Spaziano G., Mirra D., Esposito R., Altieri S., Falco G., Galoppo F.F.S., Canzano S., Sasso F.C., Matacchione G., Olivieri F., Panarese C.M.I., Paolisso P., Barbato E., Lubritto C., Balestrieri M.L., Caballero P.I.A.E., Rajagopalan S., Ceriello A., D’Agostino B., Paolisso G. (2024). Microplastics and nanoplastics in atheromas and cardiovascular events. N. Engl. J. Med..

[26] Di Fiore C., Ishikawa Y., Wright S.L. (2024). A review on methods for extracting and quantifying microplastic in biological tissues. J. Hazard. Mater..

[27] Liu S., Wang C., Yang Y., Du Z., Li L., Zhang M., Ni S., Yue Z., Yang K., Wang Y., Li X., Yang Y., Qin Y., Li J., Yang Y., Zhang M. (2024). Microplastics in three types of human arteries detected by pyrolysis-gas chromatography/mass spectrometry (Py-GC/MS). J. Hazard. Mater..

[28] Sun J., Sui M., Wang T., Teng X., Sun J., Chen M. (2024). Detection and quantification of various microplastics in human endometrium based on laser direct infrared spectroscopy. Sci. Total Environ..

[29] Salvia R., Rico L.G., Bradford J.A., Ward M.D., Olszowy M.W., Martínez C., Madrid-Aris Á.D., Grífols J.R., Ancochea Á., Gomez-Muñoz L., Vives-Pi M., Martínez-Cáceres E., Fernández M.A., Sorigue M., Petriz J. (2023). Fast-screening flow cytometry method for detecting nanoplastics in human peripheral blood. MethodsX.

[30] Geppner L., Ramer G., Tomasetig D., Grundhöfer L., Küss J., Kaup M., Henjakovic M. (2023). A novel enzymatic method for isolation of plastic particles from human blood. Environ. Toxicol. Pharmacol..

[31] Leslie H.A., van Velzen M.J.M., Brandsma S.H., Vethaak A.D., Garcia-Vallejo J.J., Lamoree M.H. (2022). Discovery and quantification of plastic particle pollution in human blood. Environ. Int..

[32] Cowger W., Steinmetz Z., Gray A., Munno K., Lynch J., Hapich H., Primpke S., De Frond H., Rochman C., Herodotou O. (2021). Microplastic spectral classification needs an open source community: open specy to the rescue!. Anal. Chem..

