# Supplementary material for: Isolation and characterization of microplastics from human blood samples by confocal RAMAN microscopy
Source: MethodsX. 2026 Feb 25;16:103841. doi: 10.1016/j.mex.2026.103841 (PMC12972724; doi:10.1016/j.mex.2026.103841)
Supplement: Supplementary file 1 — Supplementary material and/or additional information [OPTIONAL] During the optimization of the protocol, the procedure was transitioned from using larger glass fiber filters (47 mm diameter, nominal pore size (∼1.6 µm), Whatman; WHA1825047) to smaller ones with the same pore size (25 mm diameter, nominal pore size (∼1.6 µm), Whatman; WHA1820025). This reduction in filter diameter allowed a higher concentration of retained MPs, thereby improving their recovery and subsequent analysis. [file mmc1.zip › Supplemenatry caption.docx]

### **Supplementary Fig. 1. Representative examples obtained during protocol optimization.**

(A) Spectra generated under one of the digestion conditions tested (20% KOH, 1% Tween-20). Filter image (A), image of a particle selected for analysis (A′), and Raman spectrum (white) compared with reference spectra from Open Specy (red) (A′′). Although the software provided similarity with polystyrene (PS), the spectrum appeared poorly defined, suggesting that this treatment may damage the polymeric structure of MPs and compromise reliable identification. (B–D) Particles corresponding to graphite (B), carpathite (C), or potassium nitrate (D). Filter images (B–D), images of selected particles (B′–D′), and Raman spectra of the particles compared with reference spectra from Open Specy (B′′–D′′). These signals likely represent organic matter carbonized by laser exposure and were not considered microplastic particles.
